# Social Cognition Analyzer Application—A New Method for the Analysis of Social Cognition in Patients Diagnosed With Schizophrenia

**DOI:** 10.3389/fpsyt.2019.00912

**Published:** 2019-12-20

**Authors:** Eszter Varga, Róbert Herold, Tamás Tényi, Szilvia Endre, Judit Fekete, Titusz Bugya

**Affiliations:** ^1^ Department of Psychiatry and Psychotherapy, Medical School, University of Pécs, Pécs, Hungary; ^2^ Department of Paediatrics, Medical School, University of Pécs, Pécs, Hungary; ^3^ Department of Psychology, University of Pécs, Pécs, Hungary; ^4^ Department of Cartography and Geoinformatics, University of Pécs, Pécs, Hungary; ^5^ CityScience Lab, Hafencity University, Hamburg, Germany

**Keywords:** schizophrenia, social cognition, SCAN, application, response time, Scanalizer, power-law distribution

## Abstract

**Introduction:** Because of the importance of the assessment of social cognitive impairments in schizophrenia in clinical settings, a new computer application called SCAN (Social Cognition Analyzer applicatioN) was developed. Our first aim was to examine if patients diagnosed with schizophrenia could be differentiated from healthy individuals based on the results of SCAN, taking into consideration both response rates and response times. Our second aim was to create Scanalizer, as part of SCAN, to produce social cognitive profiles of individual patients.

**Materials and Methods:** 86 patients (SG) and 101 healthy participants (CG) were examined with SCAN. The domains were: ToM, irony, metaphor, emotion perception from prosody and social perception. SCAN displayed the tasks, recorded the answers and the response times. For the differentiation of the two groups a two-dimensional scatter plot was used. For the graphical presentation of the social cognitive profile of patients, the calculation of the distributions of CG’s results was made with Kolmogorov-Smirnov Goodness-of-fit Test and with the sum of squared residuals (SSR).

**Results:** We found that the SG’s response rates were significantly lower and the SG’s response times were significantly slower compared to the CG in every condition. With the two-dimensional comparison of the summary response rates and the summary response times of the participants, the SG could be differentiated from the CG and this differentiation worked irrespective of age and education. For the graphical representation of social cognitive functions of patients, distributions of the results of the CG were calculated. We found normal distributions in the response times of all conditions and in the response rates of the ToM condition. In the low-end tail of the irony condition, and in the metaphor, social perception and emotional prosody conditions, power-law distributions were found. We also found that the summary response rates of the lowest performing 10% of the CG was in the same range as the summary response rates of all examined patients.

**Discussion:** Scanalizer enables clinicians to measure and analyse social cognitive profiles of patients diagnosed with schizophrenia. Moreover, SCAN could also be used to detect social cognitive disabilities of vulnerable individuals.

## Introduction

Social cognition refers to “the mental operations that underlie social interactions, including perceiving, interpreting, and generating responses to the intentions, dispositions, and behavior of others” (1). Social cognitive impairments are remarkable in schizophrenia (2) and contribute significantly to poor overall community functioning, functional outcome of the disease and quality of life (3–5). Impairments in social cognition are also reported in first-degree relatives of patients diagnosed with schizophrenia and individuals at ultra-high risk for psychosis (6) suggesting social cognitive dysfunctions are stable traits that precede as well as predict the onset of the illness.

Schizophrenia patients show not only poorer accuracy in social cognition tasks, but it takes significantly more time for them to complete the tasks compared to healthy controls (7–10), moreover these deficits in processing speed are associated with worse global functioning (11).

Social cognition is a multifaceted process, including several domains and subdomains. In behavioral sciences, studies about social cognition with non-psychiatric individuals identify a large set of domains, self-perception, prejudice and stereotyping, empathy, hindsight bias, and counterfactual thinking, among others (12). In schizophrenia, four domains have been identified as impaired (1, 12) namely emotion processing, social perception, Theory of Mind (ToM) and attributional style. Among these, emotion processing, social perception and ToM have been found to be trait markers of schizophrenia (13–15).

*ToM* is probably the most important core component of social cognition. It is defined as the ability to attribute mental states (such as beliefs, knowledge, intentions) to the self and others (16). Several studies and meta-analyses proved that ToM is impaired in schizophrenia (14, 17).

*Emotion perception* is a lower-level sub-domain of emotion processing, which is frequently measured in schizophrenia. Identifying emotions both from faces and voices is impaired in schizophrenia (18–20).

*Social perception* refers to the ability to identify social roles, social rules, and social context (12). The identification of several interpersonal features such as relationship, intimacy, social status, and veracity is essential to deal with complex social situations. Several studies found that social perception is impaired in schizophrenia (17, 21, 22). However, Karpouzian, Alden, Reilly and Smith (23) found that high-functioning patients preserved social perception as opposed to low-functioning patients diagnosed with schizophrenia.

Social cognition is a major treatment target in schizophrenia. As antipsychotic medications were found to be ineffective in significantly improving social cognition, various trainings have been developed, specifically targeting these functions (24).

However, in order to create individualized treatment plans as well as targeted, recovery-focused and person-focused social cognitive therapeutic interventions, there is a need for a specific tool to objectively, reliably, and quickly assess social cognition of patients.

The most widely used social cognition assessments in schizophrenia are the following: the MATRICS Consensus Cognitive Battery (25), the Facial Emotion Identification Task (FEIT) (26) and The Awareness of Social Inference Test (TASIT) (27). The seventh domain of the MATRICS Consensus Cognitive Battery contains Mayer-Salovey-Caruso Emotional Intelligence Test (MSCEIT™) (25), which was developed to assess social cognition. It is a Paper-and-pencil multiple-choice test that assesses how people manage their emotions. It evaluates Emotional Intelligence (EI) through a series of objective and impersonal questions. Based on scenarios typical of everyday life, the MSCEIT measures how well people perform tasks and solve emotional problems, rather than having them provide their own subjective assessment of their emotional skills. The FEIT (26) covers the assessment of emotion perception. It is a computer based test, which involves black-and-white photographs of 19 different individuals’ faces each depicting one of six different emotions (happiness, sadness, anger, surprise, fear, shame). 15 photographs depict negative emotions (sadness, anger, fear, and shame), while 4 photographs depict positive emotions (happiness, and surprise). In the administration of FEIT, participants are required to select which of the six emotions was depicted on the picture and to mark it on a paper-based form. The Awareness of Social Inference Test (TASIT) (27) is an ecologically valid and reliable tool that assesses higher-level social perception deficits. The first part of the test assesses emotion recognition, while the second and the third part of the test assess the ability to detect literal (sincerity and lies) and non-literal (sarcasm) conversational remarks, as well as the ability to make judgments about the thoughts, intentions and feelings of speakers. Nevertheless, all of the above mentioned assessments are paper-based, requires substantial time and human resources. Moreover, their administration time is lengthy, as such, routine use of them in clinical settings is often difficult to achieve.

For all these reasons, we developed a new computer application to assess the three domains of social cognition that have been most commonly identified as impaired in schizophrenia. The application is called SCAN (**S**ocial **C**ognition **A**nalyzer applicatio**N**). SCAN is a menu-driven application with a standard graphical interactive interface. It has a user-friendly mouse management, so the respondent can complete the test independently, after getting instructions. The test operator has no other job than to start the program and to do a backup of the recorded results after the test is completed. The results of each participant’s test sessions are stored in separate folders, named after the respondent. Results from these folders can be imported into a spreadsheet for further analysis. To assess the three domains of social cognition in schizophrenia we decided to select at least one social cognitive task from each domain. We selected social cognitive tasks that are not too long, nor too complex at the same time reliable as well as sensitive enough to differentiate patients from healthy individuals (1). This selection was based on the experiments of Green et al. (12) as well as Pinkham et al. (1). The selection of pragmatic language tasks was based on the positive results of our previous experiments (25, 28–31).

Since patients with schizophrenia show deficits not only in response accuracy, but also in processing social cognitive tasks in a timely manner (7–9,, 11), SCAN measures response times along with response accuracy.

In our previous study (25), using SCAN, we found that community based psychosocial treatment had a strong influence on the social cognition of patients diagnosed with schizophrenia and a significantly positive association was found between the improvement of SCAN scores and the improvement of GAF (Global Assessment of Functioning) scores. These results might support the applicability of SCAN measuring social processing in schizophrenia.

The first aim of the present investigation was to examine if patients diagnosed with schizophrenia could be differentiated from healthy participants based on the results of SCAN, taking into consideration both response rates and response times. Our second aim was to create an application, as part of SCAN, which would be able to calculate the social cognitive profiles of individual patients. In order to present the results of individual respondents in graphical forms, we needed to calculate and graph the distributions of the response rates and the response times of the control group in every domain separately. Based on the results of previous studies (32, 33), we hypothesized that these processes would follow normal distributions.

## Materials and Methods

### Participants

86 patients with chronic schizophrenia (41 females) fulfilling the diagnostic criteria of DSM-5 were evaluated (SG). Diagnosis was confirmed by Module B and C of SCID-5 (Module B: Psychotic Symptoms, Module C: Differential Diagnosis of Psychotic Disorders) (34). Patients were recruited from the Institution of Psychiatry and Psychotherapy, University of Pécs. All of them were outpatients. Patients were on maintenance antipsychotic treatment. Necessary conditions for participation were the following: age older than 18; native Hungarian speaker; no auditory or visual impairments interfering with computer usage; no evidence of substance abuse, neurological disorder, or intellectual disability according to DSM 5; no change in the medication of the participants during the study and in the last six months prior to the study; being in the remission phase of the disease.

We obtained data for psychopathology to confirm the remission state of the patients (Positive and Negative Syndrome Scale; PANSS). It was assessed with 8 items in positive, negative and general psychopathology subscales of PANSS (P1, P2, P3, N1, N4, N6, G5, G9), which were mild or less (≤3) for at least 6 months before entering the study, according to the remission criteria of schizophrenia (35). The frequency and severity of the symptoms were evaluated by two senior psychiatrists (Herold R., Tényi T.). Inter-rater reliability was tested, and the kappa coefficient was >0,75.

The control group (CG) consisted of 101 healthy individuals (46 females). Members of the CG were recruited through online advertisement. All of them were over 18, they were native Hungarian speakers, and they had no auditory or visual impairments interfering with computer usage. They had no record of psychiatric (personal or familial) or neurological morbidity, dependence on psychoactive substances (excluding caffeine and tobacco). They were also screened with SCID. Demographic data of the two groups as well as duration of illness PANSS remission scores of the SG are shown in **Table 1**.

**Table 1:** Demographic data in the CG and the SG and PANSS total remission score in the SG.

	Control group (CG) (n = 101)		Patients diagnosed with schizophrenia group (SG) (n = 86)		
	Mean	S. D.	Mean	S. D.	p-value
Gender (female/male)	46/55		41/45		
Age (year)	37.5 (20–60)	16.16	34 (23–49)	4.24	*P* < 0.001^a^
Education (years)	19.45	10.77	11.51	1.28	*P* < 0.001^a^
Duration of illness (years)			15.02	6.3	
PANSS total remission score			15.32	2.57	

a Mann-Whitney U test was used to calculate the differences between the groups. Statistically significant: p < 0.05, uncorrected.

After complete description of the study to the subjects, written informed consents were obtained. The investigation was done following institutional guidelines. Ethical perspectives were established in accordance with the latest version of the Declaration of Helsinki. The Research Ethics Committee of the Faculty of Humanities, University of Pécs approved this study design. Participants were aware of the study aims and hypotheses.

### Social Cognition Analyzer Application

In order to assess social cognition in patients diagnosed with schizophrenia in clinical settings, an open-source psychometric software was developed, called SCAN. SCAN runs under Linux operating system, and the application itself is in Bash. It already contains a test battery (for the description of the tests see 2.3 Experimental tasks), but it can also be used as a framework program that displays text files, mp3 files, avi files, and image files in png and jpg formats.

SCAN is an easy to use program and was primarily designed for clinicians. It is a menu-driven application with a standard graphical interactive interface. It has user friendly mouse management, so after getting instructions, the respondent can complete the test independently. The test operator has no other job than to start the program and to make a backup of the recorded results after the test is completed.

After startup, SCAN asks the name, the age and the education of the respondent. After filling in personal data, the program follows with a test for checking mouse handling time, in which participants have to click on the Yes or No buttons in 9 differently sized windows, displayed after each other. By measuring mouse handling time, the respondent’s ability to use computer interface is evaluated. After that, sample tasks are displayed in order to get the respondent familiar with test types. After displaying the samples, the actual experimental tasks are displayed. The respondent only has to choose the right answer by clicking on the response window, which appears after the tasks. SCAN records the answers as well as the response times. The results of each respondent’s test sessions are stored in separate folders, named after the respondent.

The results of individual patients’ test sessions can be calculated and presented with Scanalizer (SCAN analyzer) in written as well as in graphical forms. It is important to note, that Scanalizer is only able to evaluate the results of those respondents who complete the default test battery (*Experimental Tasks*).

The framework program with its detailed manual, the Hungarian and English versions of the test battery (with the exception of the English version of the emotional prosody test) and Scanalizer can be downloaded from this website: scan.ttk.pte.hu.

### Experimental Tasks

To assess social cognition, we used five experimental domains: ToM, irony, metaphor, emotion perception from prosody and social perception. We presented 5 scenarios in the irony and 5 in the metaphor conditions, 26 tasks in the ToM condition, 24 tasks in the emotion perception from prosody condition, and 9 tasks in the social perception condition, summing up to a total of 69 tasks in our study. The tasks were introduced in a random order so as to present the different domains in an unpredictable manner.

Participants’ assessments of social cognitive functions were carried out individually in separate examination rooms in the Department of Psychiatry and Psychotherapy University of Pécs, Hungary. Testing procedures were carried out by two trained administrators (Varga E and Endre Sz). At the time of testing, the program ran on a laptop (with 15” screen). The investigation was supported by a headset and a computer mouse attached to the laptop.

### ToM

ToM is the ability to attribute mental states (such as beliefs, knowledge, intentions) to the self and others (16). We used a reduced version (26 tasks) of the “Reading the Mind in the Eyes” Test (36) to measure ToM capacity. This test requires the ability to comprehend complex mental states from eyes. In Fernández-Abascal et al. (37) they found acceptable psychometric properties in schizophrenia (test-retest reliability: r = 0.806; internal consistency: Cornbachs Alpha = 0.750). In this present study, a modified version of the test was used. For each picture, participants had to choose between two words for the best description of the mental state presented in the picture.

### Language Pragmatics

Social inferencing in language pragmatics is an important aspect of social cognition (28, 38). Pragmatics focuses not only on what people say but how they say it and how others interpret their utterances in social contexts (30). Several studies show that metaphor and irony tasks are suitable to measure pragmatic language skills in schizophrenia. As far as we know there is no study, which investigates psychometric properties of these tasks, however in our previous studies we found that patients with schizophrenia perform significantly weaker in these tasks compared to healthy controls (25, 28–31). In the present investigation, after each scenario two questions were asked concerning the figurative meaning of the metaphors and another two were asked checking the comprehension of an ironic remark in a social situation.

### Emotion Perception

Emotion perception was measured with an affective prosody test, which was designed based on the work of Edwards et al. (39). Affective prosody is the suprasegmental aspects of speech that contain emotional as well as linguistic information. An actress and an actor were asked to speak 24 (6x4) simple sentences with the appropriate affective prosody: “they must stay here”; “he will come soon”; “she will drive fast”; and “we must go there”, in the 6 basic moods in Hungarian, namely anger, sadness, happiness, disgust, fear, and surprise. Participants had to choose from two possible basic emotions which best described the feeling presented.

### Social Perception

Social perception refers to the ability to identify social roles, social rules, and social context (12). It was assessed with a movie task, which was designed based on the Interpersonal Perception Task (IPT) of Costanzo and Archer (40), in which videotaped scenes of interpersonal situations were shown. We used 9 brief scenes from different movies, each lasted 10 to 15 sec. Each of them contained one of the five common types of social judgments, such as intimacy, competition, deception, kinship, and status. Participants had to determine the correct answer by “reading” nonverbal behavior, facial expression, tone of voice, gesture, touch, glance, or hesitation (40). Test-retest reliability of the Interpersonal Perception Task was reported as 0.70 for a 5-week interval; internal reliability was reported as 0.52 (12). After each scene, participants had to choose the right answer of three possibilities.

### Statistical Analysis

We used Statistical Package for the Social Sciences (SPSS; SPSS Inc., Chicago, IL, USA; 41) version 20 for Windows, OpenOffice.org version 5.0 and Gnumeric 1.12.38 to do the statistical analysis.

In the between-group analysis, as distributions proved to be normal, independent sample t test was performed across mouse handling times, response times and ToM domain. As distributions proved not to be normal, in the demographic data as well as the irony, metaphor, emotion perception from prosody and social perception domains, Kruskall-Wallis one-way analysis of variance (ANOVA) by ranks was performed to compare group medians. As significant between-group differences were found in age and education, differences were considered in the statistical analyses, namely, two subgroups matching in age and education were created within each group and the performances of the subgroups in each domains were compared (42–45).

**Figure 1:**
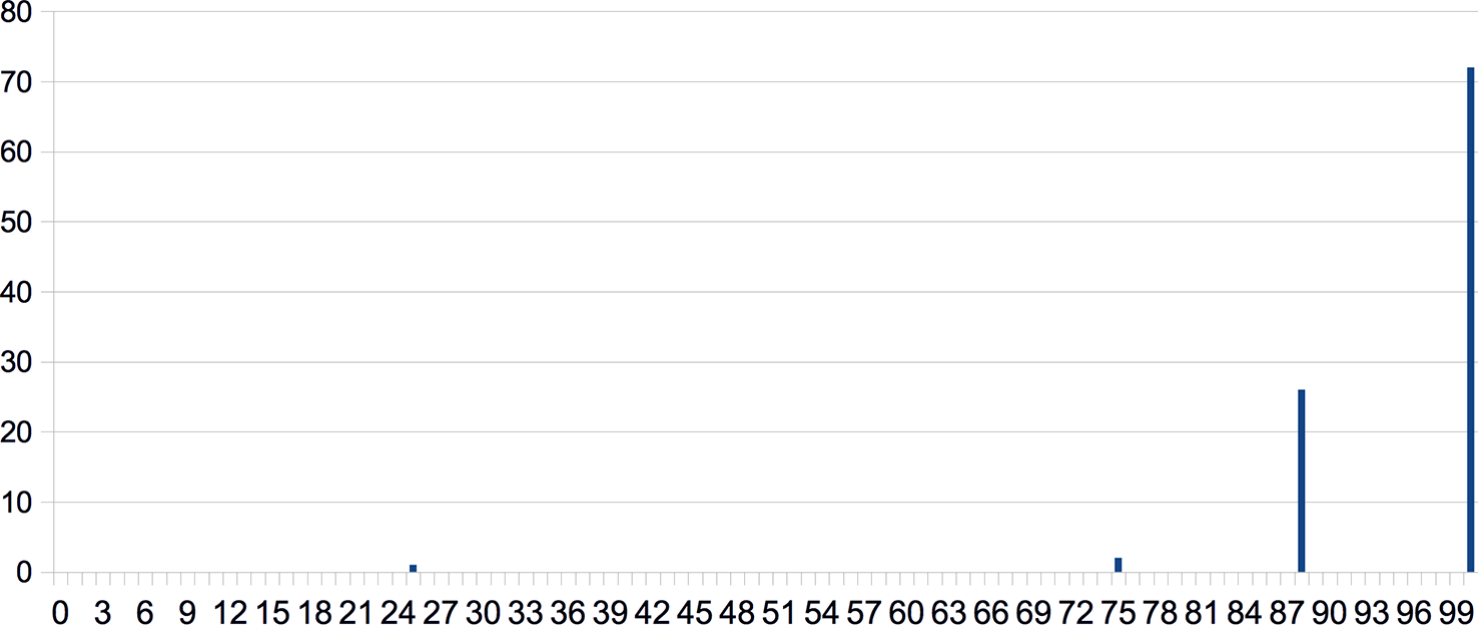
Results of the CG in the social perception condition graphed in a Cartesian coordinate system. Response rates (in %) are shown along the x-axis and ratios of those who achieved the given response rates (in %) can be found along the y axis.

For each condition the fitting of the normal, Poisson, power-law and exponential functions were measured. First, the Kolmogorov-Smirnov test was used to check normal distribution. In those cases where distributions were not proven to be normal, power-law and exponential functions were checked. Since the same method was used for each domain, the applied method was presented with the results of the prosody domain in the CG:

Results of the CG were graphed in a Cartesian coordinate system in every domain. As an example for this process, see **Figure 1**, where the ratio of those who achieved 100% was 72%, and the ratio of those who achieved 25% was 1%. **Figure 1** shows that this distribution is steeper than the normal distribution and the average is at the margin of the measured range (the theoretical maximum), instead of the mid of the distribution. Therefore, it seemed reasonable to check the fitting of power-law and exponential functions as well. The general formula of the power-law function (40, 46) is:

$$y=k\;\cdot x^{z}$$

where

*y* = calculated distribution-value;
*k* = constant value;
*x* = percent of the participants;
*z* = a constant exponent.
*k* value was specified as the maximum value of the measured distribution (decimal, between 0 and 1), and *z* value was defined as the value where this sum has the minimum value:

$$\sum_{1}^{n}{\left(a_{m}-\;a_{n}\right)}^{2}$$

*a* *_m_* – measured frequency
*a* *_n_* – calculated frequency for the same point

Using power-law distribution as a model of the measured distribution k and z parameters were defined by trial and error to reach the best fit.

The formula of the exponential function is:

$$f(x)=e^{i/100z}$$

*e* – Euler-number (∼2, 71)
*i* – current percentage class
*z* – constant multiplication factor of the exponent – definition of *z* is the same as the *z* factor of the power-law distribution (see above).

Identifying the function, which best models our data is important because these two models interpret the examined phenomena differently. At x = 0 the value of the power-law function is y = 0, consequently, the probability of the existence of an individual, who cannot interpret social cognitive tasks is zero, which, we believe, corresponds to our everyday observations. In contrast, at x = 0 the value of the exponential function is y > 0, which implies the possibility that there are individuals who cannot interpret any tasks.

To select the best fitting function, the sum of squared residuals (SSR) (47) were calculated to rule out the competing hypothesis. The presentation of the methodology is based on the data of the CG in the prosody condition (**Table 2**):

1. The measured values were recorded in a spreadsheet table. Values were recorded by 10% declaration (10% width classes), recording the number of the participants with the current percentage of correct answers. No data was recorded for the percentage-classes without measured values (see column A in **Table 2**).
2. Using the measured values, relative frequency was calculated (see column B in **Table 2**).
3. The cumulative sum (running total) of relative frequency was calculated (see column C in **Table 2**).
4. For each percentage class we also calculated the current cumulative sum value of the normal, Poisson, exponential and the power-law distribution (see column D, E, F and column G in **Table 2**).
5. The best fitting distribution – where the sum of column d_norm_, d_poisson_, d_exp_, and d_power_ is the lowest – was considered as the model of the measured values.

**Table 2:** An example for the calculation of sum of squared residuals (SSR) based on the data of the CG in the Prosody condition.

Class (percentage of correct answers)	A	B	C	D	E	F	G	H	I	J	K	L	M	N	O
–10%	0,00	0,00	0,00	0,00	0,00	1,00	0,00000	0,00	0,00	0,00	0,00	0,00	0,00	0,00	0,00
0%< – 10%	0,00	0,00	0,00	0,00	0,00	3,56	0,00000	0,00	0,00	0,00	0,00	0,00	0,00	0,00	0,00
10% – 20%	0,00	0,00	0,00	0,00	0,00	12,68	0,00000	0,00	0,00	0,00	0,00	0,00	0,00	0,00	0,00
20% – 30%	0,00	0,00	0,00	0,00	0,00	45,13	0,00002	0,00	0,00	0,00	s0,00	0,00	0,00	0,00	0,00
30% – 40%	0,00	0,00	0,00	0,00	0,00	160,69	0,00006	0,00	0,00	0,00	0,00	0,00	0,00	0,00	0,00
40% – 50%	0,00	0,00	0,00	0,00	0,00	572,12	0,00021	0,00	0,00	0,00	0,00	0,00	0,00	0,00	0,00
50% – 60%	0,00	0,00	0,00	0,00	0,00	2036,95	0,00074	0,01	0,00	0,00	0,00	0,00	0,00	0,00	0,00
60% – 70%	0,00	0,00	0,00	0,00	0,01	7252,33	0,00264	0,02	0,01	0,00	0,01	0,00	0,00	0,00	0,00
70% – 80%	5,00	0,05	0,05	0,01	0,11	25821,08	0,00941	0,08	0,06	0,01	0,07	0,00	0,00	0,00	0,00
80% – 90%	26,00	0,26	0,31	0,34	0,44	91932,96	0,03349	0,28	0,22	0,04	0,28	0,00	0,02	0,00	0,00
90% –	70,00	0,69	1,00	0,92	0,81	327316,61	0,11926	1,00	0,69	0,11	1,00	0,01	0,04	0,00	0,00
**Sum**												**0,0089**	**0,0575**	**0,0021**	**0,0011**

**A**, Measured distribution (number of test in the given class); **B**, Measured distribution in the percentage of the total number of test; **C**, Cumulative sum of column **B**; **D**, Values of the calculated (fitted) normal distribution; **E**, Values of the calculated (fitted) Poisson distribution; **F**, Values of the calculated (fitted) exponential distribution; **G**, Normalized values column F; **H**, Cumulative values of column G; **I**, Values of the calculated (fitted) power-law distribution; **J**, Normalized values column I; **K**, Cumulative values of column J; **L**, Sum of squared residuals (SSR) for the normal distribution; **M**, Sum of squared residuals (SSR) for the Poisson distribution; **N**, Sum of squared residuals (SSR) for the exponential distribution; **O**, Sum of squared residuals (SSR) for the power-law distribution.

## Results

### Demographic Characteristics

We found significant between-group differences in age (*z* = -7,801, *p* < 0,001) and in the years of education (*z* = -8,533, *p* < 0,001). The demographic data of the two groups are summarized in **Table 1**.

### Performance in Domains, Mouse Handling Times, and Response Times

We found that the SG was not significantly slower in mouse handling time than the CG (t = 0.982, *p* = 0.328 n.s.; **Table 3**).

**Table 3:** Differences in social cognition task performance (%) and response time (sec) between CG and SG.

	Control group (CG)		Patients diagnosed with schizophrenia group (SG)		
	Mean	S. D.	Mean	S. D.	p-value
*Mouse Handling Time*	3.11	2.05	3.66	1.57	*P* = 0.329^b^
***Social Cognition***	93.20	8.69	72.83	17.41	*P* < 0.001^a^
*Theory of Mind*	85.68	8.70	74.88	12.88	*P* < 0.001^b^
*Metaphor*	96.92	7.93	69.99	25.35	*P* < 0.001^a^
*Irony*	95.34	12.14	58.74	18.31	*P* < 0.001^a^
*Social Perception*	95.67	9.34	81.94	17.00	*P* < 0.001^a^
*Emotional Prosody*	92.40	5.51	78.60	13.24	*P* < 0.001^a^
***Response Time***	5.87	1.92	8.18	5.21	*P* < 0.001^a^
*Theory of Mind*	7.30	2.44	9.49	7.36	*P* < 0.001^b^
*Metaphor*	6.31	2.22	9.34	5.24	*P* < 0.001^b^
*Irony*	3.54	1.3	6.84	4.71	*P* < 0.001^b^
*Social Perception*	9.02	2.54	15.01	6.04	*P* < 0.001^b^
*Emotional Prosody*	3.76	1.24	5.51	2.55	*P* < 0.001^b^

a Mann-Whitney U test was used to calculate the difference between the groups.

b Independent sample t test was used to calculate the difference between the groups.

Statistically significant: p < 0.05, uncorrected.

The CG performed significantly more accurately in all the domains (eyes test: *z* = -6,458, *p* < 0,001; metaphor: *z* = -7,864, *p* < 0,001; irony: *z* = -11,089, *p* < 0,001; social perception: *z* = -6,004, *p* < 0,001; emotional prosody: *z* = -7,164, *p* < 0,001) than the SG. These between-group differences still remain significant after the Bonferroni correction (*p* < 0,01; **Table 2**).

We found that the SG was significantly slower in all domains (eyes test: *t* = 4,342, *p* < 0,001; metaphor: *t* = 5,521, *p* < 0,001; irony: *t* = 5,879, *p* < 0,001; social perception: *t* = 9,069, *p* < 0,001; emotional prosody: *t* = 6,094, *p* < 0,001; **Table 2**).

Because of the significant age and educational differences between the groups, two subgroups matching in age and education were created within each group. This is a frequently used method in the literature of social cognition research in schizophrenia, however it is usually used when IQ scores of the groups taking part in the experiment differ significantly (42–45). Firstly, the highest education score in the SG and the lowest education score in the CG were used as lower and upper thresholds: individuals with an education score higher than 5 as well as those with lower than 3 were removed from each group. Data of the remaining subgroups were further analyzed, namely the highest age in the SG and the lowest age in the CG were used as lower and upper thresholds: individuals with an age score higher than 47 as well as those with lower than 25 were removed from each group. Data of the matched subgroups were further analyzed. The patients subgroup contained 52 individuals (24 females) (mean education = 3.6, SD = 1.03; mean age = 35.2, SD = 4.1) and the control subgroup contained 34 individuals (15 females) (mean education = 4.1, SD = 0.6; mean age = 36.4, SD = 10.08). We found no significant difference in age (*z* = -1,756, *p* = 0,082 n.s.) and in the years of education (*z* = -1,842, *p* = 0,069 n.s.) between the remaining SG and CG.

The SG subgroup still performed significantly worse in all the domains than did the age and education matched healthy controls (eyes test: *z* = -4,679, *p* < 0,001; metaphor: *z* = -5,902, *p* < 0,001; irony: *z* = –7,354, *p* < 0,001; social perception: *z* = –2,942, *p* = 0,003; emotional prosody: *z* = –5,742, *p* < 0,001). These between-group differences still remained significant after the Bonferroni correction (*p* < 0,01). In addition, the matched SG’s response times were significantly slower in all the domains as well (eyes test: *t* = 1,319, *p* = 0,004; metafora: *t* = 3,351, *p* = 0,005; irony: *t* = 4,852, *p* < 0,001; social perception: *t* = 2,843, *p* = 0,020; emotional prosody: *t* = 2,121, *p* = 0,013).

The summary (average) social cognition response rate of the SG was significantly lower (z = –6,085, *p* < 0,001), and the summary response time of the SG was significantly slower (t = 6.692, *p* < 0,001) compared to the CG (**Table 3**). In the matched subgroups the summary social cognition response rate of the SG was significantly lower (z = –5,191, p < 0,001), and the summary response time of the SG was significantly slower (t = 3.092, *p* = 0,002) compared to the CG.

**Figure 2:**
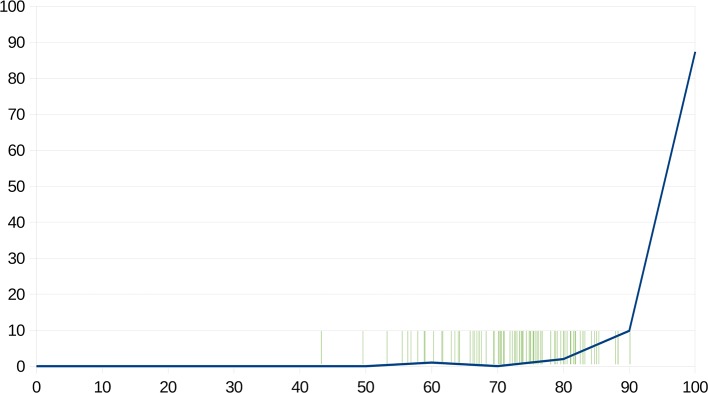
The blue line shows what percentage of the CG achieved a certain percentage of summary response rate. The green lines on the horizontal axis indicate the summary social cognitive response rate of every patient in the study. X-axis: Response rates (in %); Y-axis: ratios of those who achieved the given response rates (in %).

In **Figure 2**, we illustrated the response accuracy of the CG as a whole compared to the response accuracy of the patients individually. The blue curve shows what percentage of the CG achieved a certain percentage of summary task performance. The green lines on the horizontal axis indicate the summary social cognitive response rate of every single patient in the study. The figure shows that the patients’ performances were between 43% and 90%, and the worst performing 10% of the CG was in the same range as the patients.

**Figure 3:**
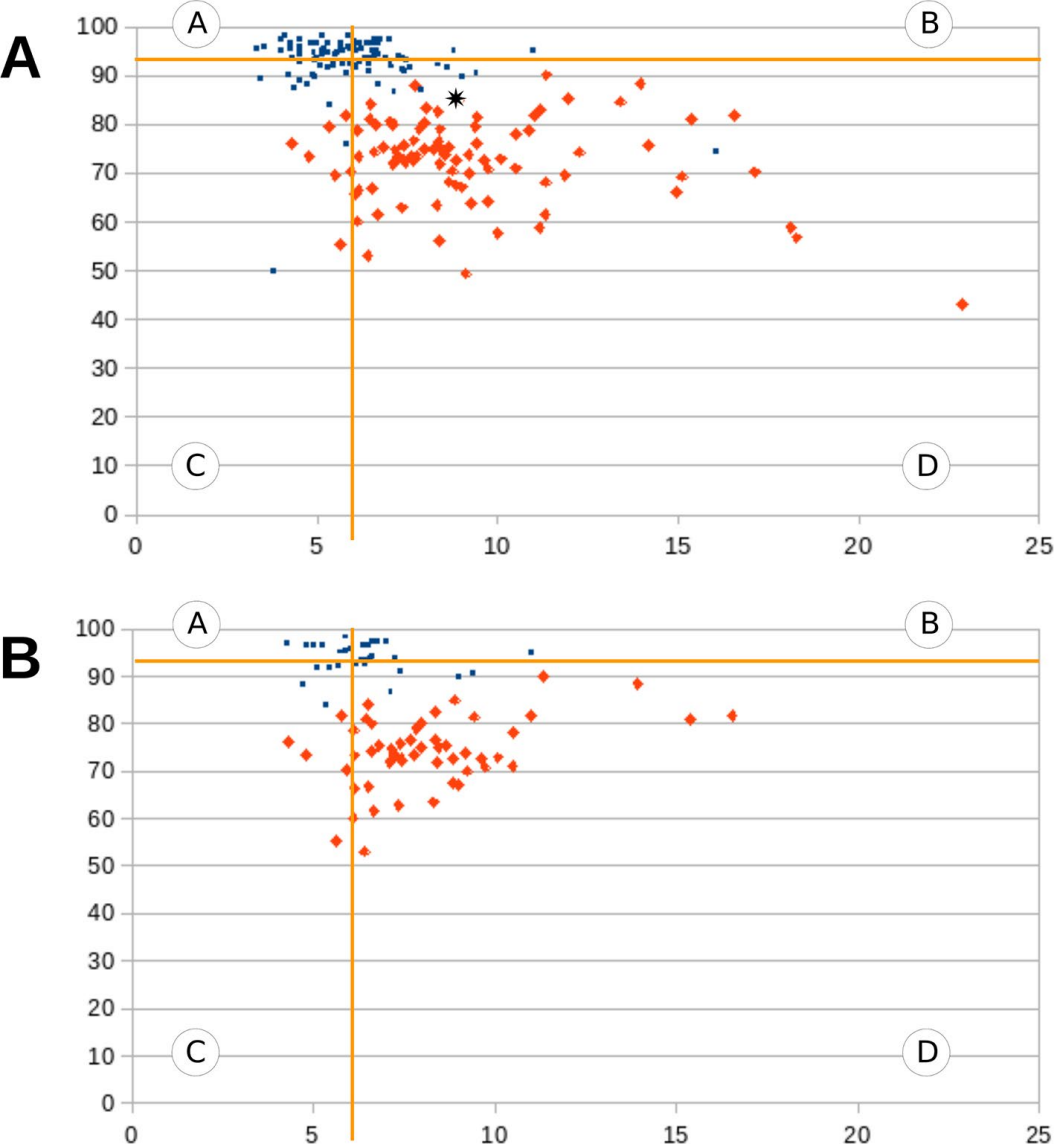
**(A**, **B)** Graphical differentiation of the CG and the SG (**Figure 3A**), as well as the two matched subgroups (**Figure 3B**). Each participant’s social cognitive performance was symbolized by a single point defined by their summary response time (along the x-axis) and summary response rate (along the y-axis). The blue dots represent healthy participants and the red dots represent schizophrenic participants. The diagrams were divided into four fields by a horizontal and a vertical line. The horizontal line represents the mean response rate of the CG, and the vertical line represents the mean response time of the CG.

In order to answer the question whether we would be able to distinguish patients diagnosed with schizophrenia from healthy controls, based on the results of SCAN, a two-dimensional comparison was performed, taking into consideration both response rates and response times. Thus, two-dimensional scatter plots were created for the graphical differentiation of the two groups (**Figure 3A**), as well as the two matched subgroups (**Figure 3B**) in terms of their social cognition. In **Figures 1B** and **Figure 3A**, each participant’s social cognitive performance was symbolized by a single point defined by their summary response time (along the x-axis) and their summary response rate (along the y-axis). The diagrams were divided into four fields: the horizontal line represents the mean response rate of the CG, and the vertical line represents the mean response time of the CG. Each field represents different types of performances:

**A**–more correct answers than the mean of the controls, and shorter response time than mean of the controls;
**B**–more correct answers than the mean of the controls, and longer response time than mean of the controls;
**C**–less correct answers than the mean of the controls, and shorter response time than mean of the controls;
**D**–less correct answers than the mean of the controls, and longer response time than the mean of the controls.

### The Operation of Scanalizer

**Figure 4:**
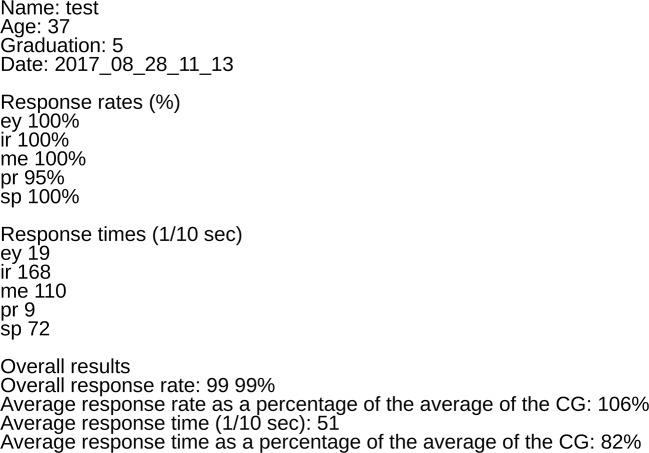
The figure shows an example text file with the overall results of a participant created by Scanalizer.

To achieve our second aim, Scanalizer was designed to analyze and present social cognitive characteristics of a single patient with schizophrenia by comparing his/her results with the results of the CG. Scanalizer produces three types of results for each respondent: a text file with the overall results (**Figure 4**), as well as two types of graphical results. In the graphical representation of the results Scanalizer uses a two-dimensional scatter plot (described in the last paragraph) for the estimation of the social cognitive performance of a patient (**Figure 3A**). **Figure 3A** shows how Scanalizer presents the overall result of a selected patient from the SG with a black mark.

**Figure 5:**
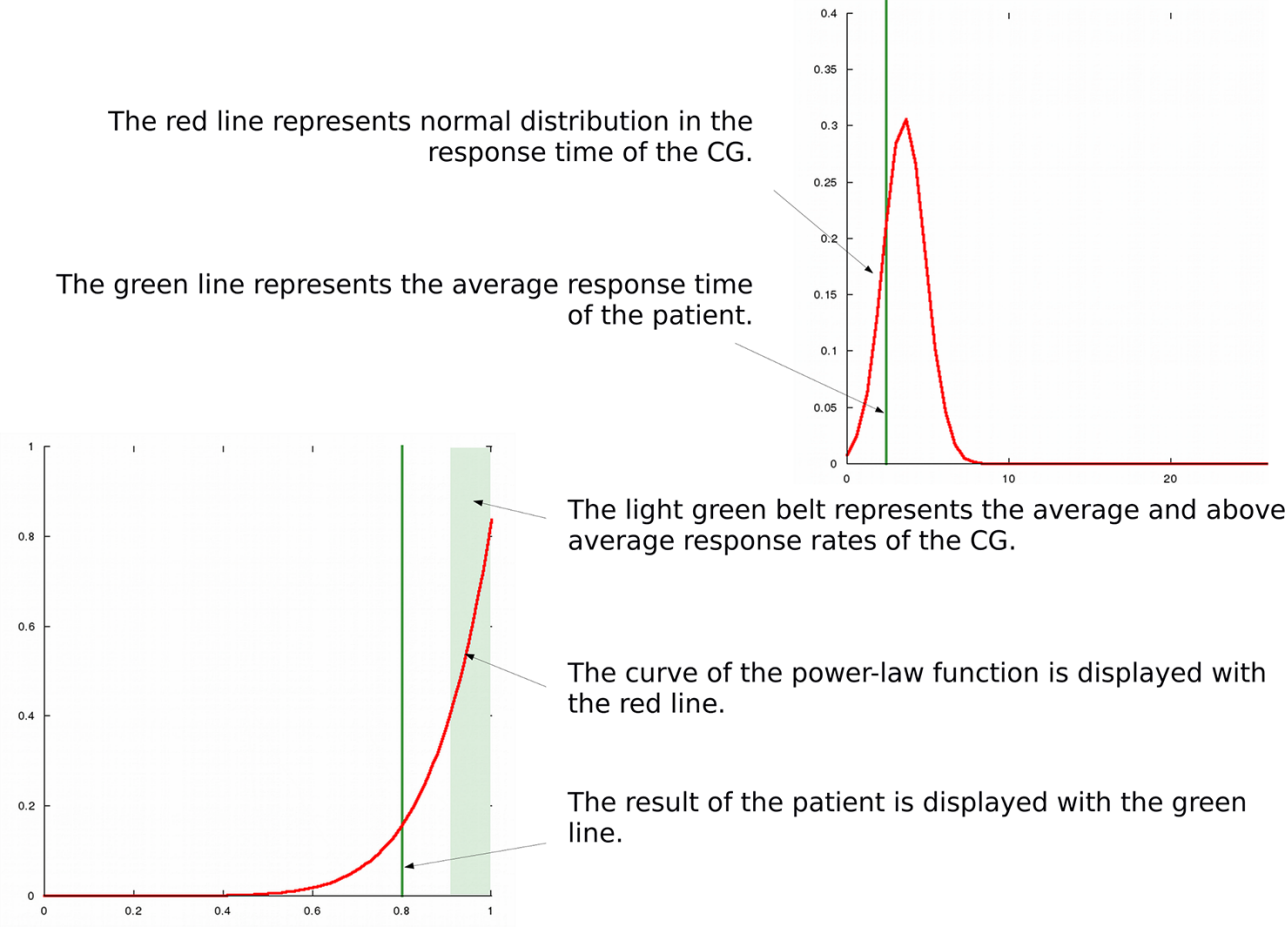
Graphical results of a patient in the irony condition displayed by Scanalizer.

Scanalizer is also able to graphically display the response rates and the response times of a patient in every domain separately. For this purpose, we needed to calculate and graph the distributions of the response rates and response times of the CG in every domain separately. These graphs serve as the base graphs for Scanalizer to graphically render the results of individual respondents. For example, in **Figure 5** shows the graphical representation of the patient’s results in the irony condition.

### Distributions of Data

**Figure 6:**
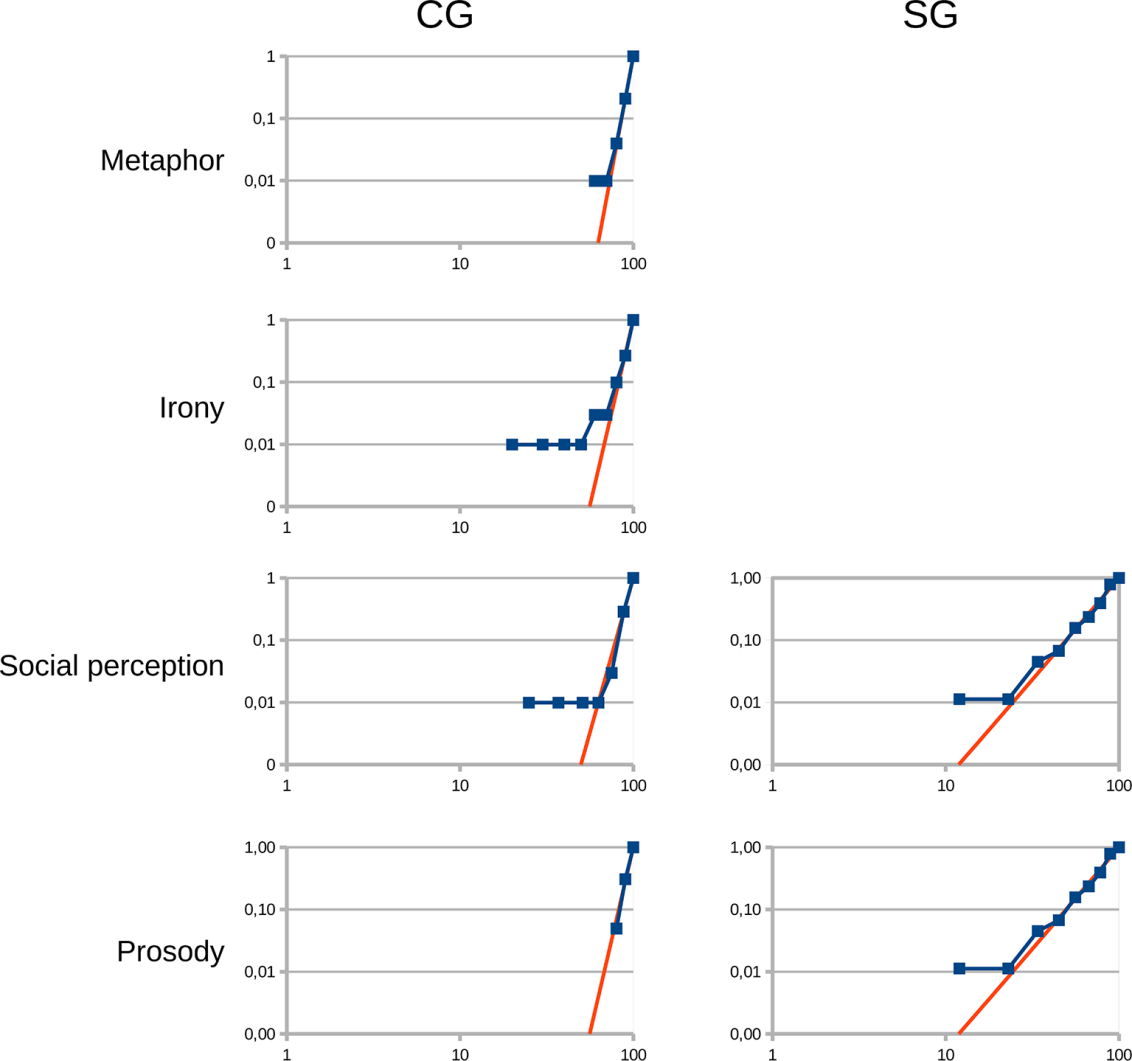
Power-law distributions in the irony, metaphor, emotional prosody and social perception conditions in the control group (CG) and in the patients diagnosed with schizophrenia group (SG). X-axis: Response rates in %; Y-axis: Cumulative ratios of those who achieved the given response rates (ratio from 0 to 1).

Regarding the response rates, normal distributions were found in the ToM condition in the CG, as well as in the ToM, irony and metaphor conditions in the SG. At the same time, power-law distributions were found in the metaphor, social perception and emotional prosody conditions in the CG, and in the social perception and emotional prosody conditions in the SG. In the CG, however, exponential distribution was found in the irony condition (z = 12.7). While further analyzing the data we found that at the low-end tail (to 10%) the power-law distribution provided good fits of the data (**Figure 6**). Distribution types, SSR values as well as *k* and *z* values in the CG and in the SG are summarized in **Table 4**.

**Table 4:** Constant value (k), constant exponent (z) from the formula of the power-law distribution (*y* = *k* · *x* *^z^*) and sum of squared residual (SSR) values (*d* *_exp_* *and d* *_power_*) in the CG and in the SG.

domains	Control group (CG)				Patients diagnosed with schizophrenia group (SG)			
	*k*	*z*	*d* *_exp_*	*d* *_power_*	*k*	*z*	*d* *_exp_*	*d* *_power_*
*Metaphor*	0.75	12	0.0004	0.0002	-	-	-	-
*Irony (low-end tail)*	0.73	11	6.72·10^-11^	2.59·10^-24^	-	-	-	-
*Social Perception*	0.71	9	0.0018	0.0011	0.39	2.3	0.8258	0.0149
*Emotional Prosody*	0.69	11	0.0021	0.0011	0.36	3.3	1.1647	0.0480

Response times in all domains showed normal distributions in the CG as well as in the SG.

## Discussion

The first aim of our present investigation was to see if it is possible to distinguish patients diagnosed with schizophrenia from healthy individuals based on the results of SCAN, by taking into consideration both response rates and response times. Our other aim was to design an application, which is able to calculate and present the social cognitive profile of a single patient. For graphical representation, we needed to calculate the distributions of the response rates and response times of the CG in every condition.

Our results showed that we could differentiate patients diagnosed with schizophrenia from healthy individuals based on the results of SCAN, with a two-dimensional estimation, and this differentiation worked irrespective of age and education (**Figures 3A, B**). Regarding the distributions of the CG, as we had expected, we found normal distributions in the response times in all domains as well as in the response rates of the Eyes Test. However, in the metaphor, social perception and emotional prosody tasks, as well as in the low-end tail of the distribution of the irony tasks, power-law functions provided good fits of the data.

SCAN was primarily designed for clinicians to assess social cognition objectively and reliably in patients diagnosed with schizophrenia.

We believe, that SCAN has substantially more advantages compared to the most commonly used social cognitive assessments (25–27). SCAN is able to measure the most widely investigated dysfunctional domains of social cognition in schizophrenia (1, 12) in one setting. In our test battery, social cognitive tasks were varied, including scenarios, pictures, sounds and short movie scenes and were presented randomly in order to model real life social situations more closely. The administration time of SCAN is relatively short, which allows its routine use in clinical settings. The important advantages of SCAN over paper-based tests are that patients are able to complete it without considerable assistance and that results can be analyzed within a few minutes, so any patient’s social cognitive profile is available in a very short time. Note, that SCAN can also be used as a framework program that displays any text files, mp3 files, avi files, and image files in png and jpg formats, which means that the investigated social cognitive domains can be modified optionally.

Response rates and response times were both taken into consideration when examining if the interpretation of the tasks were processed in a timely manner. We need to point out that measuring response times is highly important in order to better understand the dynamics of social processing both in healthy individuals and in patients diagnosed with schizophrenia. For example, understanding a joke at a party while having a conversation with a couple of people means not only understanding the intended meaning of the humorous utterance, but also understanding it in time, otherwise the situation can be missed, because conversations move on fast.

In line with our present findings, several studies found significant impairments in ToM, emotion perception and social perception in schizophrenia (2, 20, 48, 49), as well as in response times in tasks requiring social cognitive abilities (7–9, 10, 11). However, our study is the first one that took into consideration both response rates and response times for the representation of social cognitive performance in patients diagnosed with schizophrenia. In **Figures 3A, B** blue dots represent social cognitive performance of the CG, and red dots represent social cognitive performance of the SG. These graphs clearly distinguish the SG from the CG, as controls are mainly located in fields A or B, and patients are exclusively located in fields C or D. As the social cognitive performance of the SG was significantly worse compared to the CG both in the non-matched as well as in the age and education matched groups, we propose that SCAN is able to detect impaired social functioning of patients diagnosed with schizophrenia irrespective of sociodemographic characteristics.

SCAN also contains a set of assessments to measure mouse handling time. With mouse handling time, we intended to measure participants’ reaction time when answering questions with a computer mouse. Since impaired reaction time has been found in patients diagnosed with schizophrenia (50, 51), measuring mouse handling time is important for the correct judgment of response times in computer based cognitive tasks. In our present study, mouse handling time of the SG did not differ significantly from that of the CG, therefore we propose, that the significant differences found in response times between the two groups show real response time differences in social cognitive tests.

As far as we know, SCAN is the first computer tool, with which clinicians can calculate and present social cognitive functions of individual patients diagnosed with schizophrenia. This process can be done with the help of the application called Scanalizer, which is part of SCAN. Scanalizer produces three types of results: a text file with the overall results (**Figure 4**), as well as two types of graphical results. One provides an overall picture of the social cognitive performance of the patient (**Figure 3A**), and the other presents the response rates and the response times of the respondent in each domain separately, graphed on the density functions of the CG’ data (**Figure 5**).

For the latter, it was necessary to calculate the distributions of the response rates and the response times of the healthy participants in every domain separately. As for the results, normal distributions were found in the response times in all domains as well as in the response rates of the Eyes Test. Contrary to our expectation, in the metaphor, social perception and the emotional prosody tasks, power-law functions provided good fits of the data with the exponents of 9 (social perception), 11 (prosody) and 12 (metaphor). In several empirical phenomena, the low-end tail of the distributions follow power-law (52) as we found in the irony tasks. The distribution of the irony tasks has exponential form at the high-end.

Many measurements of living systems cluster around the average. When a cognitive process follows normal distribution, only a negligible amount of probability is far from the average, making the average representative of the process. As the central limit theorem shows, the production of normal distribution is the combination of random and independent effects. However, as researchers in cognitive science have found in recent decades, not all distributions fit this pattern, although, many processes obey scaling laws (53–58). Correspondingly, Kello et al. (46) pointed out that living systems are more than collections of random and independent effects, and that the existence of scaling laws in cognitive sciences describe a fundamental order in living systems.

In our data, power-law distributions show that in the CG, the probability of the existence of individuals with 100% performance is the highest and the probability of the existence of individuals with lower performances is rapidly decreasing. In power-law distributions, contrary to normal distributions, the probability of unusual events occurring simultaneously with usual events is relatively high. Consequently, there is a relatively high probability of healthy individuals with the unusually low social cognitive performances, who present together with individuals with the usual 100% performances. Moreover, power-low distributions at the low-end tails show that the probability of the existence of an individual, who cannot interpret any social cognitive tasks is zero, which corresponds to our everyday observations.

Shannon’s information theory (59) studies the quantification, storage, and communication of information. Its impact is crucial to the studying of linguistics and communication. Ferrer i Cancho posits in his communication model (60) that the goal of communication is to maximize the information transfer as well as to save the cost of the signal use. As the perception of social cognitive signals is an important part of human communication it is obvious that this phenomenon is also present in the processing of many social cognitive tasks in the present study. We found that various values of the exponents can be detected in the different domains, which might depend on the weight of the information transfer in the tasks (60). Moreover, the cost saving feature of social processing might give rise to errors in the comprehension of social cognitive tasks, in a certain percentage of individuals.

It is well known that the comprehension of many social signals is highly context dependent (29, 61, 62). Accordingly, when less contextual information is available, the probability of making errors during the interpretations increases (63). At the same time, when there is not enough contextual information, the available meaning will be the one which occurs at the highest frequency in social situations, similarly to the word frequency effect (64).

In Ferrer i Cancho’s communication model (60), the exponent of the laws grows as the weight of the information transfer increases in the communication. Accordingly, we suggest that power-law distributions with 9≤z ≤ 12 means that social signals in the experimental tasks have highly clear meaning in the given contexts, and the exponents might grow if the availability of the contextual information increases or if the tasks contain high frequency social signals.

In contrast, the results of the Eyes Test in the CG follow a remarkably different pattern, namely normal distribution. This result is consistent with the results of previous studies (62, 65, 66). According to the communication model and the frequency effect mentioned above, we propose that there are two main reasons for this result: one might be the low availability of sufficient contextual information (as mental states have to be judged only from eye-region expressions), and the other might be that some of the used mental terms have relatively low frequency in usual social situations. Thus, when completing the Eyes Test, the information transfer between social signals and meanings cannot be maximized. The explanation may be that this is an advanced test that has been developed for people living with high-functioning autism in order to eliminate the use of compensatory strategies (33).

Response rates of the SG in the Eyes test also followed normal distribution, however, as opposed to the CG, normal distributions were found in the response rates in the metaphor and irony tasks, i.e. in pragmatic language comprehension. We propose that this remarkably different pattern of comprehension in the population of patients diagnosed with schizophrenia might support pervasive communicative-pragmatic difficulties in them (67).

Similarly to the CG, the SG also showed power-law distributions in the prosody and in the social perception tasks with lower exponents, 3.3 (prosody) and 2.3 (social perception). According to the communication model described in the previous paragraphs, we propose that the results of the SG indicate that in patients diagnosed with schizophrenia the processing of the context and/or the ability to select the proper meaning of a social signal among competing meanings are impaired (29, 66, 68–71). Furthermore, the lower exponents in the prosody and social perception tasks might show the upset of the balance between maximizing the information transfer and saving the cost of the signal use (60).

To summarize, our results showed that in the healthy population, the comprehension of social cognitive tasks, which include sufficient contextual information and/or include social signals that have higher availability in everyday social interactions follow power-law distributions. This means that the vast majority of healthy individuals interpret these tasks correctly, however, there are also those who do not fully understand them. According to Davis et al. (72), having subclinical social disabilities is a potential vulnerability factor for schizophrenia. As illustrated in **Figure 2**, the summary social cognitive response rates of the worst performing 10% of the CG is in the same range as the summary social cognitive response rates of every examined patient (43–90%). Interestingly, several studies reported the prevalence of schizotypal traits and the prevalence of vulnerability factors for schizophrenia between 5–10% (73) and 4–15% (74, 75) in the general population. A review of Pearlson and Folley (76) emphasized that single endophenotypic abnormalities in the healthy population can be in the range of 15%–20%.

## Conclusions

As far as we know, SCAN is the first computer tool for clinicians to objective, reliably, and quickly assess social cognition in patients diagnosed with schizophrenia. Based on our results with SCAN, we could clearly distinguish patients from healthy individuals in their social cognitive performances, when we took into consideration both response rates and response times. Moreover, with the help of Scanalizer, clinicians are able to measure and analyse social cognitive profiles of patients diagnosed with schizophrenia.

Our results also suggest, that SCAN may also be suitable to detect individuals with subclinical social difficulties.

Another important conclusion of our study is that the response rates of the CG in various social cognitive tasks follow power-law distributions, which suggest a fundamental order in social cognitive task processing. Whether power-law distributions could be detected in data obtained from real social interactions may be a topic for further investigations.

## Limitations

Our study has several limitations. First of all, there were significant differences in age and education between the CG and the SG. Even though, we compared the age and education matched subgroups, we still found significant differences in the investigated variables.

Another limitation is that we did not investigate the effect of basic neurocognition and IQ on social cognitive performance because it was out of the scope of our study as our main aim was to assess the applicability of SCAN in a patient sample living with schizophrenia. We also did not have data on medical treatment history, treatment compliance or the use of inpatient and outpatient services of the patients. Nonetheless the demographic data of the SG (age, education, and illness length) suggest that our sample represents a population of patients with chronic course of schizophrenia. This population usually conceived as an important target of psychosocial interventions aiming to improve social cognition, and hence a better outcome of the disorder. The feasibility of this approach is partly supported by our earlier data on the effect of social interaction on social cognition in a very similar patient population (77). However it would be important that future studies address these questions, as the relationships between these variables are rather controversial. The lack of psychiatric control group as well as the lack of providing PANSS scores are further limitations of our study.

Our results concerning the distributions in social cognitive processing of healthy individuals should be interpreted cautiously since the sample size is rather low and not representative. Thus, further investigations as well as the replication of the present results are needed.

## Data Availability Statement

All datasets generated for this study are included in the article/supplementary material.

## Ethics Statement

After complete description of the study to the subjects, written informed consents were obtained. The investigation was done following institutional guidelines. Ethical perspectives were established in accordance with the latest version of the Declaration of Helsinki. The Research Ethics Committee of the Faculty of Humanities, University of Pécs approved this study design. Participants were aware of the study aims and hypotheses.

## Author Contributions

EV: study design, stimulus construction, data collection, data analysis, writing article, manuscript revision. RH: stimulus construction, writing article, manuscript revision. TT: stimulus construction, writing article, manuscript revision. SE: data collection, writing article, manuscript revision. JF: data collection, writing article, manuscript revision. TB: software development, data analysis, writing article, manuscript revision.

## Funding

This research project was supported by the KTIA-13-NAP-A-II/12 (2018–2022) and the Hungarian National Excellence Centrum Grant 2018–2019.

## Conflict of Interest

The authors declare that the research was conducted in the absence of any commercial or financial relationships that could be construed as a potential conflict of interest.
